# Perillyl alcohol, quercetin, and berberine combination therapy ameliorates experimental pulmonary arterial hypertension: Effects on the lung miR-204 expression, remodeling, and inflammatory factors

**DOI:** 10.22038/AJP.2024.24522

**Published:** 2024

**Authors:** Zeinab Kordestani, Ahmad Beik, Hamid Najafipour, Zohreh Safi, Majid Askaripour, Soodeh Rajabi

**Affiliations:** 1 *Physiology Research Center, Institute of Neuropharmacology, Kerman University of Medical Sciences, Kerman, Iran *; 2 *Gastroenterology and Hepathology Research Center, Institute of Basic and Clinical Physiology Sciences, Kerman University of Medical Science, Kerman, Iran*; 3 *Department of Physiology and Pharmacology, Afzalipour Medical Faculty, and Physiology Research* *Center, Kerman University of Medical Sciences, Kerman, Iran*; 4 *Endocrinology and Metabolism Research Center, Kerman University of Medical Sciences, Kerman, Iran*; 5 *Department of Physiology, School of Medicine, Bam University of Medical Sciences, Bam, Iran*; 6 *Cardiovascular Research Center, Institute of Basic and Clinical Physiology Sciences, Kerman University of Medical Science, Kerman, Iran*

**Keywords:** Pulmonary artery hypertension, Perillyl alcohol, Berberine, Quercetin, Combination therapy, MiR-204

## Abstract

**Objective:**

Pulmonary artery hypertension (PAH) is a devastating syndrome. Our previous studies showed that perillyl alcohol (P), berberine (B) and quercetin (Q) improve PAH. In this study, we investigated the effects of sub-effective doses of these derivatives in double and triple combination forms on PAH in rats.

**Materials and Methods:**

Forty nine rats were divided into seven groups (n=7): 1) control, 2) monocrotaline (MCT), 3) MCT+vehicle (veh), 4) MCT+BP, 5) MCT+PQ, 6) MCT+BQ, and 7) MCT+BPQ. After three weeks of PAH induction with MCT (60 mg/kg), either vehicle (ethanol 5% in saline) or one of the above combinations (dose of 20 mg/kg for B, and doses of 20 and 10 mg/ kg for P and Q in vehicle) was administered for three weeks. Right ventricular (RV) pressure, contractility indices, lung pathology, miR-204 expression, oxidative stress markers, inflammation and apoptosis were assayed.

**Results:**

MCT increased RV systolic pressure and hypertrophy, and lung arteriole wall thickness, fibrosis and leukocyte infiltration in the MCT group compared to the CTL group. All treatments improved these effects significantly. Furthermore, MCT reduced antioxidant factors, Bax, P21 and miR-204 expression and increased Tumor Necrosis Factor alpha (TNF-α), Interleukin 6 (IL-6) and Bcl-2. All of these effects were recovered by combination treatments.

**Conclusion:**

The results showed that combination therapy with sub-effective/ineffective doses of these compounds had ameliorative effects against PAH.

## Introduction

Pulmonary artery hypertension (PAH) is a devastating syndrome with a poor prognosis. The principal characteristic of PAH is the remodeling of the pulmonary vessels and a progressive rise in its vascular resistance and pressure, leading to right ventricle (RV) hypertrophy and eventually premature death due to RV failure (Handoko et al., 2010). The vascular remodeling consists of medial hypertrophy/hyperplasia, intimal and adventitial fibrosis, plexiform and thrombotic lesions, and perivascular infiltration of various types of inflammatory cells such as macrophages, B and T lymphocytes, dendritic cells, and mast cells (Sommer et al., 2021)_._

Although the approved therapies have somewhat increased the survival rate of PAH patients, they mainly exert their effects by reducing vascular tone (Humbert et al., 2023). However, it is now accepted that curative approaches must not only dilate the vessels but also target vascular remodeling. 

The prevention and treatment of diseases by natural products have a long history, and many drugs in modern medicine are also extracted from natural sources (Bahar et al., 2007; Newman et al., 2016). Berberine (B), quercetin (Q), and perillyl alcohol (P) are herbal ingredients with numerous properties, including anti-inflammatory and antioxidant effects (Rajabi et al., 2021). In our previous studies, we have shown that each of these three substances can treat PAH and reduce related complications (Rajabi et al., 2020; Rajabi et al., 2021; Beik et al., 2023). 

This study examines the effects of double and triple combinations of these substances on PAH. In combination therapy, lower doses are used, which can lead to avoidance or reduction in side effects, although therapeutic effectiveness is still achieved (Tallarida et al., 2010). 

Small RNAs including miRNAs, are short, non-coding nucleotide sequences that have recently been shown to involve in many physiological and pathological processes (Vishnoi et al., 2017). miR-204 is one of miRNAs involved in PAH. It has been found that miR-204 decreases pulmonary artery smooth muscle cells (PASMCs) in human and rats and reduction of miR204 expression activates proliferative and anti-apoptotic pathways (Src–STAT3–NFAT pathway) in PAH (Courboulin et al., 2011). Our recent studies have shown that Q, and P were independently able to affect the expression of miR-204 and its targets, HIF1α and NFATc2 (Rajabi et al., 2020), as well as miR-29a and miR-33a (Rajabi et al., 2022) in the lungs to reduce inflammation and oxidative stress. Also, Q, P and B reduced oxidative stress, inflammation, fibrosis and apoptosis in the heart of PAH rats via affecting miR-204 (Rajabi et al., 2021)^.^ . We hypothesize that combination of small doses of these compounds that were ineffective alone may improve lung and heart complications of PAH effectively. In the present study, the combined double or triple doses of these three herbal ingredients were examined to evaluate their possible ameliorative effects on the complications of PAH, and on the expression of miR-204 in the lung and hearts of rats.

## Materials and Methods

Male Wistar rats were obtained from Animal Center of Kerman University of Medical Sciences, Iran. All animal experiments and protocols were approved by the local ethical committee of Kerman University of Medical Sciences (IR.KMU.REC.1397.606). Wistar rats (240-280 g) were kept in standard conditions (12 hr light/dark cycle and 23±2°C temperature) to adapt with environmental conditions for one week. 

Ketamine and xylazine hydrochloride (Bremer pharma, Germany), B, Q and P (Sigma Aldrich, Japan), monocrotaline (MCT) (Sigma Aldrich, China), antibodies (Santa Cruz, USA), miRNA extraction kit (Biobasic, Canada), and ELISA kits (Biotech, China) were used in this study.

### Animals and treatments

Forty-nine rats were divided into seven groups (n=7 each): 1) control (CTL), 2) MCT, 3) MCT + Veh., 4) MCT + BQ, 5) MCT + QP, 6) MCT + BP, and 7) MCT + BPQ. 

PAH induction was performed according to the protocol published before (Rajabi *et al.* 2020). After three weeks of MCT injection (induction of the disease), the animals received vehicle (ethanol %5 in physiological saline) or double combination of B20 mg/kg + Q20 mg/kg, Q20 mg/kg+P20 mg/kg, B20 mg/kg+P20 mg/kg, or triple combination of B20+P10+Q10, once a day by intra-peritoneal (I.P.) injection for three weeks. Optimum effective dose of Q, B and P when administered alone in related dose-response studies was found to be 30 mg/kg for Q, 30 mg/kg for B and 50 mg/kg for P (Rajabi et al., 2020; Rajabi et al., 2021). In the present study, the lowest effective dose of B and ineffective doses of Q and P (according to the previous dose-response studies) (Beik et al., 2023; Rajabi et al., 2020; Beik et al., 2021), were used. Therefore, for double combination therapy, the dose of each compound was 20 mg/kg. For triple combination (BPQ group), B 20 mg/kg, and the ineffective doses of Q and P (both 10 mg/kg), were used. Therefore, a total dose of 40 mg/kg was used in both double and triple combinations. The mortality and survival rate of animals were also recorded during the study.

### Hemodynamic evaluations

At the end of study (day 43), the animals were anesthetized by ketamine and xylazine (80 and 10 mg/kg, ip, respectively). Tracheotomy was performed in order to ventilate the animal if necessary (rate of 70/min and volume of 1.5 ml /100 grams of body weight) (MA01746, Harvard Apparatus, USA). For recording systemic arterial blood pressure (SBP), a polyethylene catheter was inserted into the left carotid artery and connected through pressure transducer to a Physiograph (Powerlab, ADInstrument, NSW, Australia). Another catheter was inserted into the RV (through the jugular vein) and the right ventricular systolic pressure (RVSP) was recorded as an indicator of pulmonary artery pressure. Finally, the chest was opened and the heart and lungs were removed, weighed and rapidly frozen in -80^o^C. Right ventricular hypertrophy index (RVHI) calculated by the following formula: [RV weight/ (LV+S) weight] where LV is for left ventricle and S is for septum.

### Histopathology of the lungs 

A part of the lungs was fixed in 10% formalin for 48 hr and then cut into 5-μm sections. For assessment of pulmonary fibrosis, Masson's-trichrome staining and for assessment of arterial wall thickness, congestion and leukocyte infiltration, Hematoxylin-Eosin staining was used. Ten arteries with external diameter of 50 to120 µm in each section were evaluated for wall thickness (WT) (computer-light microscope, Olympus, Japan). 

The formula used for %WT was: [(external diameter - internal diameter)/external diameter] ×100 (Cai et al., 2019). 

The scoring system for quantification of fibrosis, congestion and infiltration was from 0 to 4 as follows: normal = (0), up to 25% (1), 26-50% = (2), 51-75% = (3) and 75- 100% = (4). For assessment of these criteria, 6 fields were observed in each slide. 

### Western blotting 

The lung tissue samples were homogenized (Hielscher homogenizer, ultrasound technology, Germany) in lysis buffer (RIPA buffer). Then, the homogenates were centrifuged (13000 rpm at 4°C for 15 min). The supernatant (protein) concentration was assayed through a Nanodrop (ND2100, Thermo Fisher Scientific, Waltham, MA). The supernatant samples containing 40 µg of protein were loaded on 12.5 % sodium dodecyl-sulfate polyacrylamide gel electrophoresis (SDS-PAGE) and transferred onto the poly vinylidene fluoride (PVDF) membrane.

Following a blocking by non-fat powdered skimmed milk, the PVDF membrane was incubated with primary antibodies against Bax, P-21, and Bcl-2, for 5 hr then incubated for 3 hr with goat anti-rabbit IgG H&L (HRP) secondary antibodies at room temperature. The protein bands were made visible by advanced chemiluminescence (ECL) and they were detected by the Bio-Rad Gel Doc/Chemi Doc imaging system. Image J software was used to assay the intensity of band densities of protein. β-actin protein was used as a housekeeping protein for loading (Askaripour et al., 2022).

### ELISA

Tumor necrosis factor alpha (TNF-α) and interleukin 6 (IL-6) were measured with ELISA commercial kits in lung tissue (Biotech, China). Different standard solution concentrations were made by using the standard source, then 50 µl of them was added to wells of the plate. In the next step, 40 μl sample, 10 μl primary antibody, and 50 µl streptavidin- horse radish peroxidase )HRP( were loaded into the sample wells. The plate was incubated for 1 hr at 37°C, then the plate was washed and loaded with 50 μl chromogen A and B respectively. After 10 min incubation, 50 μl stop solution was added to the wells of the plate, and absorbance was evaluated using an ELISA microplate reader (EPOCK, USA) at 450 nm (Askaripour et al., 2023).

### Oxidative stress indicators

A part of lung tissue was used to evaluate oxidative stress. Catalase activity was determined by Sinha's protocol. At first, H_2_O_2_, potassium phosphate, and sodium phosphate buffer were added to the supernatant samples, then acetic acid and potassium dichromate were added, and incubation was done at 100 ° C for 10 min. The solution was centrifuged at 2500 rpm for 5 min and the absorbance was assayed at 570 nm (Weydert and Cullen, 2010). glutathione peroxidase (GPx) activity was assayed by Paglia's protocol. Glutathione, glutathione reductase, NADPH, and H_2_O_2_ were added to the samples. At the end, the absorbance was assessed at 340 nm (Paglia and Valentine, 1967).

The Randox kit protocol was used for the measurement of super oxide dismutase (SOD) activity. At first, the samples were combined with xanthine, phosphate buffered saline (PBS) and xanthine oxidase. Optical density was determined at 505 nm at min 1 and 5 (Weydert et al., 2010). 

Malondialdehyde (MDA) was measured as follows: 200 µl thiobarbituric acid and trichloroacetic acid reagent were added to the samples and then incubated for 1 hr at 100 °C. The samples were cooled and centrifuged at 1000 rpm for 10 min. Optical density was read at 535 nm.

The FARP method was used for total antioxidant capacity (TAC) activity. Acetic acid, sodium acetate, tripyridyltriazine (TPTZ), and FeCl_3_ were added to the samples. In the next step, the samples were incubated at 37^o^C for 5 min, and absorbance was assessed at 593 nm (Benzie and Strain, 1996).

### MiRNA extraction and real time PCR

MiR-204 expression was evaluated by real-time PCR technique. Here, 50 mg lung and heart tissues were homogenized (ultrasonic homogenizer, UP 200H, Germany), and RNA was extracted based on the instruction of the total RNA Mini Prep Kit (Bio Basic, Canada). The Takara Bio, Japan RT Kit was used for the synthesis of cDNA from RNA. miRNA expressions were evaluated using Amplicon master mix green high Rox (Ampliqune - Denmark) and miRNA-specific stem-loop primer using a step one plus real-time PCR system (Applied Biosystem, USA) (Jafarinejad-Farsangi et al*.*, 2015) . The U6 was used as internal control. Table 1 demonstrates the primer sequences used for miR-204 expression assay. The fold change method according to cycle threshold (CT) value was used for investigating themiR-204 expression. Fold change of gene expression levels in treatment groups versus control groups was calculated by the formula: ΔCT = CT target gene – CT control gene, Fold change = 2^–ΔΔCT^ (Amirkhosravi et al., 2023). 

### Statistical analysis

SPSS version 18 software was used for analysis. Data are expressed as mean±SEM. The normality distribution of the data was determined by the Shapiro-Wilk test, and one-way ANOVA was used to explore the differences among the groups, followed by Tukey’s post hoc test for between-group comparisons. p values of <0.05 were considered significant.

## Results

### Effect of combination therapy on RVSP and RVHI

**Figure 1:**
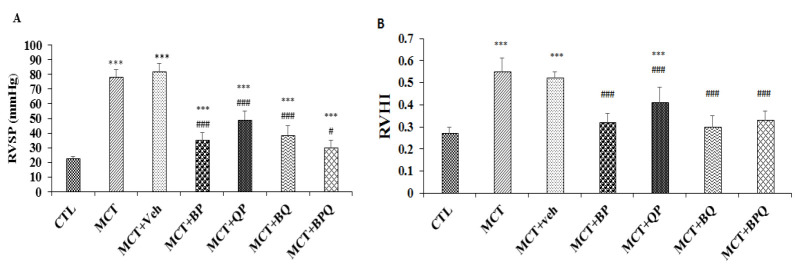
Right ventricular systolic pressure (RVSP) (A), and right ventricular hypertrophy index (RVHI) (B) values in different groups. n=7, ***p<0.001 vs the control group, ###p<0.001 and #p<0.05 vs the MCT+Veh. group. Data are presented as mean±SEM. RVSP, Right ventricular systolic pressure; RVHI, right ventricular hypertrophy index; CTL, control; MCT, monocrotaline; Veh, vehicle; BP, Berberine +Perillyl alcohol; QP, Quercetine + Perillyl alcohol; BQ, Berberine + Quercetine; BPQ, Berberine +Perillyl alcohol+ Quercetine.

On day 43 after MCT injection, RVSP increased to 77 mmHg. Also, a significant increment in RVHI happened in the PAH (MCT) group relative to the control group (p<0.001 for all) (Figure 1A and B). Treatment with vehicle did not affect PAH. Double and triple combinations of BP, QP, BQ and BPQ reduced RVSP to less than 50 mmHg which was significantly different from the vehicle group (p<0.001 for all). Also, these treatments reduced RVHI compared with the vehicle group (p<0.001 for all). 

### Effect of combination therapy on other hemodynamic and morphometric indices

In Table 2, the effects of combination treatments on other hemodynamic and morphometric indices are shown. Systolic arterial pressure was not significantly different among the groups. RV end diastolic pressure (RVEDP), maximum rate of increase in systolic pressure (+ dp/dt max) and maximum rate of reduction in ventricular pressure (-dp/dt max) and RV pressure time index (RVPTI) in the MCT and vehicle groups increased significantly. 

For most indicators, combination treatments significantly corrected these alterations although in some cases such as RVEDP, only BPQ was effective and in -dp/dt max index, QP treatment did not have a significant effect. Also, right ventricular contractility index (RVCI) decreased in PAH rats and BP and BPQ treatments increased it significantly (Table 2). 

### Effect of treatments on lung histopathology

**Figure 2:**
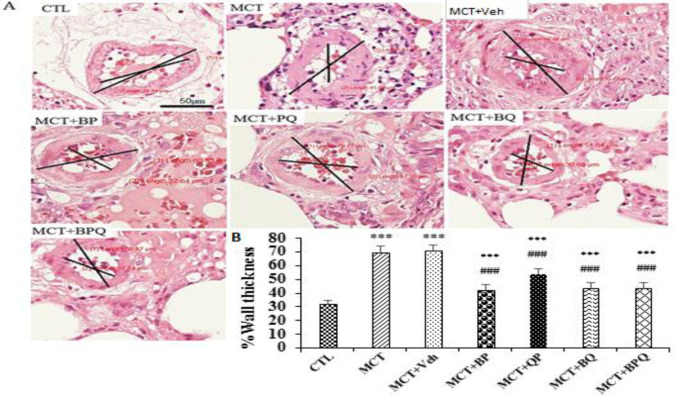
Schematic presentation of artery remodeling in lung tissue of rats. External diameter of arterioles assessed was in the range of 50–120 µm. A: Histopathology images after H&E staining (Magnification ×800) and B: Quantitative analyses of wall thickness in the studied groups. Ten arteries in each rat, n=7. Scale bar is 50 µm. Data are presented as mean±SEM. ***p<0.001 vs the control, and ###p<0.001 vs the MCT +Veh. CTL, control; MCT, monocrotaline; Veh, vehicle; BP, Berberine +Perillyl alcohol; QP, Quercetine + Perillyl alcohol; BQ, Berberine + Quercetine; BPQ, Berberine +Perillyl alcohol+ Quercetine.

Monocrotaline with a dose of 60 mg/kg and after 42 days led to several pathological changes in the lung tissue of rats. One of these changes was vascular remodeling that led to an increase in the wall diameter of the lung arterioles and a narrowing of the lumen (Figure 2). 

**Figure 3:**
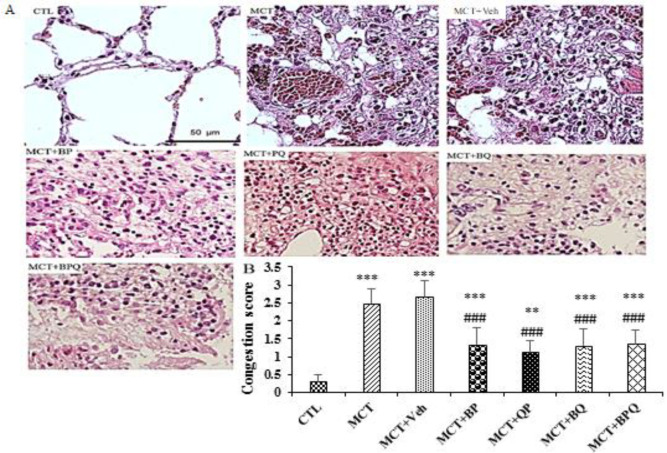
Congestion and infiltration in the studied groups. A: Histopathology images after H&E staining and B: Relative quantitative analyses in experimental groups (six fields in each sample slide), n=7 rats. Scale bars are 50 µm. Data are presented as means±SEM. ***p<0.001 and **p<0.01 vs the control, and ###p<0.001 vs the MCT+Veh. CTL, control; MCT, monocrotaline; Veh, vehicle; BP, Berberine +Perillyl alcohol; QP, Quercetine + Perillyl alcohol; BQ, Berberine + Quercetine; BPQ, Berberine +Perillyl alcohol+ Quercetine.

**Figure 4:**
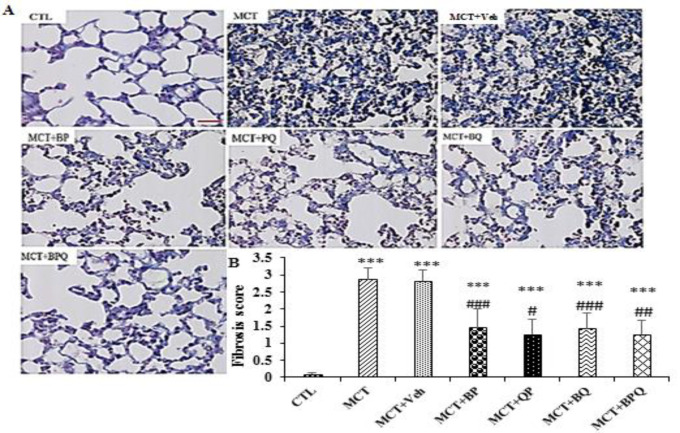
Fibrotic alterations (Masson trichrome staining) in lung tissue in the studied groups. A: Histopathology images. B; Quantitatification of fibrosis in different groups (6 fields in each sample slide), n=7 rats. Scale bar is 20 µm. Data are presented as mean±SEM. ***p<0.001 vs the control, and #p<0.05, ##p<0.01, and ###p<0.001 vs the MCT+Veh. CTL, control; MCT, monocrotaline; Veh, vehicle; BP, Berberine +Perillyl alcohol; QP, Quercetine + Perillyl alcohol; BQ, Berberine + Quercetine; BPQ, Berberine +Perillyl alcohol+ Quercetine.

All of 4 combination doses were able to significantly reduce the thickness of pulmonary arterioles (p<0.001 for all). Also, the amount of fibrosis and inflammation increased in the lung tissue in PAH rats (Figures 3 and 4). All treatments reduced these indices to a mild level.

### Effect of combination therapy on oxidative stress

**Figure 5:**
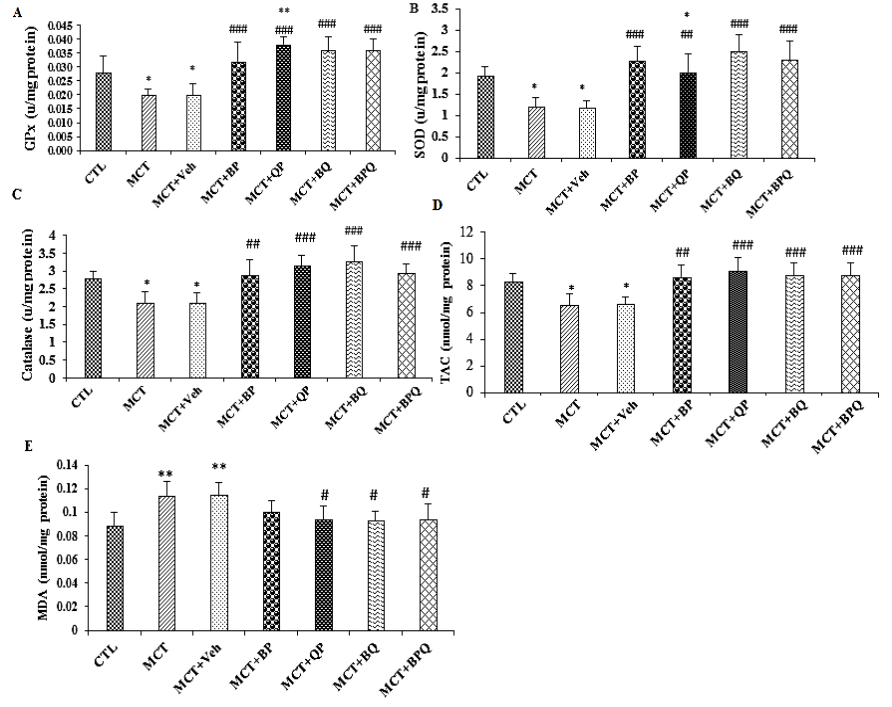
Alterations of oxidative stress indicators in the lung of different groups. Glutathione peroxidase (A), superoxide dismutase (B), catalase (C), TAC (D) and MDA (D). The data are presented as means±SEM. n=7 rats, **p<0.001 and *p<0.01 vs the control and #p<0.05, ##p<0.01, and ###p<0.001 vs the MCT+Veh. group. GPx, glutathione peroxidase; SOD, super oxide dismutase; TAC, total antioxidant capacity; MDA, Malondialdehyde; CTL, control; MCT, monocrotaline; Veh, vehicle; BP, Berberine +Perillyl alcohol; QP, Quercetine + Perillyl alcohol; BQ, Berberine + Quercetine; BPQ, Berberine +Perillyl alcohol+ Quercetine.

PAH induction by MCT reduced the antioxidants GPx, SOD, and catalase and TAC and increased MDA level in the lung tissue of rats with PAH (Figure 5). Three weeks of treatment with BP, QP, BQ and BPQ recovered the level of antioxidants to the control level. Also, treatments reduced MDA level although the effect of BP combination was not significant compared to MCT+Veh group in this case.

### Effect of combination therapy on inflammation

**Figure 6:**
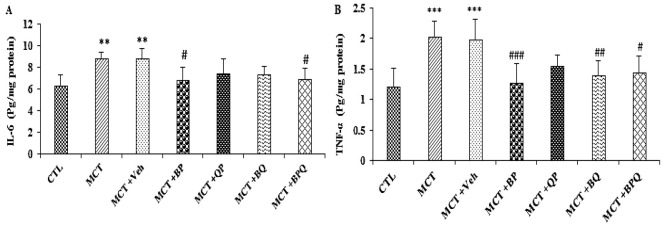
Effect of combined treatments on inflammatory cytokines IL-6 (A) and TNF-α (B) in the lung tissue of the study groups. n=7 rats. **p<0.01 and ***p<0.001 vs the Control, and #p<0.05, ##p<0.01 and ###p<0.001 vs the MCT+Veh. TNF-α, tumor necrosis factor alpha; IL-6, interleukin 6; CTL, control; MCT, monocrotaline; Veh, vehicle; BP, Berberine +Perillyl alcohol; QP, Quercetine + Perillyl alcohol; BQ, Berberine + Quercetine; BPQ, Berberine +Perillyl alcohol+ Quercetine.

With induction of PAH by MCT, the expression of IL-6 and TNF-α increased in the lungs of PAH rats compared with the CTL group significantly (Figure 6). BP and BPQ treatments reduced IL-6 level compared to MCT+Veh group significantly (p<0.05). The effect of the other two treatments was not significant compared to MCT+Veh group. Also, the level of TNF-α decreased after treatment with BP, BQ and BPQ compared to MCT+Veh group (p<0.05 to p<0.01).

### Effect of combination therapy on apoptosis and cell cycle

**Figure 7:**
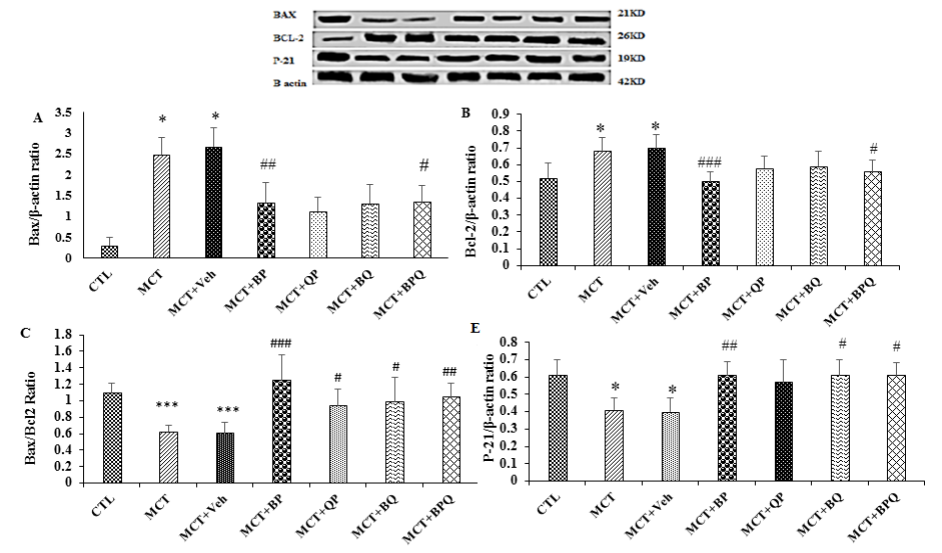
Effect of combined treatments on the markers of apoptosis in the lung of the studied groups. The expression of Bax (A), and Bcl-2 (B), Bax/Bcl2 ratio (C) and P-21 (D). Data are presented as mean±SEM (n=7 rats). *p<.05 and ***p<.001 vs the control, and #p<0.05, ##p<0.01 and ###p<0.001 vs the MCT+Veh. CTL, control; MCT, monocrotaline; Veh, vehicle; BP, Berberine +Perillyl alcohol; QP, Quercetine + Perillyl alcohol; BQ, Berberine + Quercetine; BPQ, Berberine +Perillyl alcohol+ Quercetine.

MCT decreased the expression of Bax (pro-apoptotic protein) and P-21 (cycle cell inhibitory protein), and increased the Bcl-2 (anti-apoptotic protein) in the lungs of PAH rats (Figure 7). Three weeks of treatment with BP and BPQ recovered Bax and Bcl-2 compared to MCT+Veh group significantly (p<0.05 to p<0.001). All treatments reduced Bax/Bcl-2 ratio. Also, BP, BQ and BPQ caused significant increase in P21 level compared to MCT+Veh group. 

### The effect of treatments on miR-204 in the lung and heart

**Figure 8:**
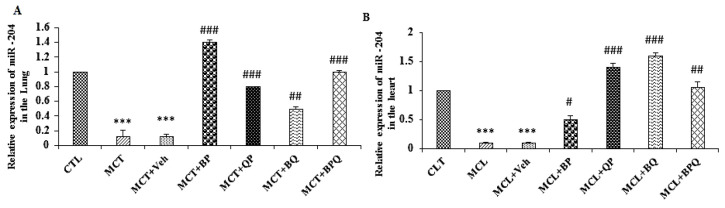
Alterations in miR-204 expression (relative to U6) in the lung (A) and heart (B) of PAH rats and the effect of combination treatments on it. Data are presented as means±SEM (n=7 rats). ***p<0.001 vs the control, and #p<.05, ##p<0.01 and ###p<0.001 vs the MCT+Veh. CTL, control; MCT, monocrotaline; Veh, vehicle; BP, Berberine +Perillyl alcohol; QP, Quercetine + Perillyl alcohol; BQ, Berberine + Quercetine; BPQ, Berberine +Perillyl alcohol+ Quercetine.

miR-204 expression decreased in the heart and lung of rats with PAH compared to CTL group (p<0.001). Treatment with BP, QP, BQ and BPQ increased miR-204 expression both in lung and heart compared to the MCT and MCT-vehicle groups, significantly (Figure 8).

### Effects of combination therapy on animal survival

No mortality was observed in the control, BP and BPQ groups until the end of the study. In the MCT and vehicle groups, four animals (57%) died in the fourth to sixth weeks. In the QP group, two animals (28%) died in the sixth week, and in the BQ group, one animal (14%) died in the sixth week. These animals were replaced, so that at the end of the study there were 7 animals in each group.

## Discussion

The results of this study showed that treatment with double or triple combinations of perillyl alcohol (P), berberine (B) and quercetin (Q) in the rats with PAH ameliorated the elevated levels of RVSP and ventricular hypertrophy and remodeling in pulmonary arteries. In parallel with these changes, indices of RV contractility such as ± dp/dt max and RVPTI improved in most treatment groups, indicating improved right ventricular function. All treatments also reduced fibrosis, leukocyte infiltration, pulmonary congestion, and mortality rate. Based on the biochemical findings, it seems that these protective effects were exerted by suppressing inflammatory responses, strengthening the antioxidant/oxidant balance, reduction of proliferation and recovery of miR-204 expression.

In our previous studies, we investigated the effect of monotherapy with Q, B and P on PAH. These plant substances were able to reduce RVSP and hypertrophy and other adverse cardiac effects of PAH (Rajabi et al., 2020; Rajabi et al., 2021; Beik et al., 2023). In the present study, the effect of combination therapy with these substances was investigated for the first time. Although the doses used were ineffective or had suboptimum effects in monotherapy, now the combinations appeared quite effective on disease complications. It seems that these compounds may amplify each other's effects on PAH because their single doses have been sub-effective/ineffective. Although the probable side effects of the compounds have not been assessed in our studies, but the small doses of the three substances applied in the combination treatment, will rule out possible side effects that these compounds may have due to larger doses used in monotherapy method.

So far, combination therapy has been used to treat some diseases. For example, the combination of some prostanoids with some endothelin receptor antagonists have had better effects than monotherapy in improving the function of patients with PAH (Lajoie et al., 2017). New guidelines suggest combination treatment instead of monotherapy due to a lower risk for patients, except for cardiopulmonary comorbidities and the elderly (Rahaghi et al., 2023). Also, in the treatment of systemic hypertension, combination therapy with two or three drugs, which have different mechanisms of action, has shown better results than monotherapy in controlling blood pressure and reducing vascular events in the heart and brain (Guerrero et al., 2018). In addition to the greater and faster effectiveness of combination therapy due to the involvement of different signaling pathways, researchers believe that the drugs used in combination therapy may also neutralize some of the each others' side effects (Guerrero et al., 2018).

Overall, the results showed that double and triple combination of Q, P and B reduced the lung artery wall thickness, fibrosis, congestion, leukocyte infiltration, and pulmonary artery pressure, and consequently resulted in an improvement in right ventricular hypertrophy. Since much lower doses of compounds were administered in combination therapy compared to monotherapy, it can be concluded that these herbal compounds may amplify each other's effects against PAH. Therefore, these compounds may be introduced as therapeutic goals towards complementary studies for the production of medicines for the treatment of heart and lung complications of PAH.

**Table 1:** Primer sequencing of miR-204 and RNU6

miR-204	RT	5′-GTTGGCTCTGGTGCAGGGTCCGAGGTATTCGCACCAGAG CCAACAGGCAT-3’	
	Forward	5′-GCGGCGGTTCCCTTTGTCATCCT-3’	
	Reverse	5′- GTGCAGGGTCCGAGGT-3’	
RNU6	Forward	5′-CTCGCTTCGGCAGCACA-3’	
	Reverse	5′-AACGCTTCACGAATTTGCGT-3	

**Table 2:** Hemodynamic and morphometric measurements in MCT-induced PAH, and the effect of combination therapies.

	**CTL**	**MCT**	**MCT+Veh**	**MCT+BP**	**MCT+QP**	**MCT+BQ**	**MCT+BPQ**
SBP (mmHg)	104±11	103±8	107±10	103±11	103±8	99±8	104±9
RVEDP (mmHg)	0.53±0.5	3.65±1.2^**^	3.15±1.5^**^	1.5±0.9^*^	1.1±7.4	0.9±3	0±1.7^#^
RV +dp/dt max (mmHg/s)	726±112	1696±185^***^	1715±298^***^	1050±356^##^	1512±348^***^	1062±313^###^	987±316^###^
RV-dp/dt max(mmHg/s)	-495±152	-1771±347^***^	-1723±298^***^	-898±284^##^	-1152±186	-850±144^##^	-695±176^###^
RVPTI (mm Hg.s)	2±0.43	8.1±7.6^***^	7.1±9.8^***^	4.0±4.8^*###^	4.0±8.5^###^	4.0±8.5^**###^	4.0±7.8^###^
RVCI (s^-1^)	86±25	51±19^**^	56±21^*^	83±12^#^	75±17	65±17	83±14^#^

SBP, systemic blood pressure; RVEDP, right ventricular (RV) end-diastolic pressure; RV +dp/dt max, dt maximum rate of rise in RV pressure; RV -dp/dt max, maximum rate of reduction in RV pressure; RVPTI, RV pressure time index; RVCI, RV contractility index. Data are as mean±SEM. CTL, control; MCT, monocrotaline; Veh, vehicle; BP, Berberine +Perillyl alcohol; QP, Quercetine + Perillyl alcohol; BQ, Berberine + Quercetine; BPQ, Berberine +Perillyl alcohol+ Quercetine. *p<0.05, **p<0.01 and ***p<0.001 vs the control, #p<0.05, ##p<0.1, and ###p<0.001 vs the MCT+vehicle.
